# Feasibility Study for the Quantification of Fullness and Discomfort in the Chest and Hypochondrium

**DOI:** 10.3390/jcm14134465

**Published:** 2025-06-23

**Authors:** Keun Ho Kim, Jeong Hwan Park, Seok-Jae Ko, Jae-Woo Park

**Affiliations:** 1 Digital Health Research Division, Korea Institute of Oriental Medicine, Daejeon 34054, Republic of Korea; 2 Korean Medicine Data Division, Korea Institute of Oriental Medicine, Daejeon 34054, Republic of Korea; 3 College of Korean Medicine, Kyung Hee University Hospital at Gangdong, Seoul 05278, Republic of Korea

**Keywords:** abdominal examination, abdominal algometric pain, abdominal shape, physical metrics, fullness and discomfort

## Abstract

**Background/Objective:** Abdominal examination by medical doctors is undertaken to observe abdominal shape and tenderness, but it is not typically quantified. Our goal was to explore the potential of physical metrics for identifying significant differences between individuals with fullness and discomfort in the chest and hypochondrium (FDCH) and those without FDCH. We utilized a 3D camera and a digital algometer to obtain these metrics. **Methods**: We screened sixty participants with functional dyspepsia and complaints of epigastric discomfort or pain and sixty healthy participants without any digestive problems as a case-control study. We assessed the degree of agreement with FDCH of the abdominal signs diagnosed by traditional East Asian medicine doctors by performing clinical studies that involved assessing abdomens with the aforementioned devices. **Results**: Algometric features such as pressure, depth, and stiffness (defined as the pressure-to-depth ratio) were significantly lower in the FDCH group than in the non-FDCH group, with mean differences across locations ranging from −1.47 to −0.86, −8.75 to −4.46, and −0.31 to −0.12, respectively. Therefore, the physical algometric features decreased, the skin stiffness decreased, and the sensitivity increased. The point estimates for the mean differences in the geometric factor of depth between FDCH and non-FDCH across the locations ranged from −2.09 to −1.66, with generally smaller depth values in the FDCH group, indicating a flat or drooping abdominal shape. **Conclusions**: The algometric and geometric metrics showed differences between the FDCH and non-FDCH groups, and various physical metrics will be expanded to identify other diseases through the collection of more clinical data in future. Trial registration/Protocol registration: CRIS and KCT0003369.

## 1. Introduction

As the elderly population grows, interest in health is also increasing, leading to greater focus on both functional and organic diseases. Since the abdomen houses vital organs, various symptoms often manifest in this area. Abdominal examinations (AEs) in traditional East Asian medicine (TEAM) are essential for diagnosing conditions such as tumors and identifying abnormalities in the intestines. These examinations assess symptoms like stiffness, tenderness, and geometric proportions of the abdomen. AEs serve as standard diagnostic tools for abdominal signs and pattern identification (PI), ensuring accurate treatment. Additionally, AEs allow both doctors and patients to directly assess the abdominal condition and track disease progression [1,2].

Functional dyspepsia (FD) is a common functional gastrointestinal disorder characterized by upper abdominal symptoms without any identifiable structural cause. According to the Rome III criteria, FD is defined as the presence of one or more of the following symptoms: bothersome postprandial fullness, early satiation, epigastric pain, or epigastric burning, with no evidence of structural disease to explain the symptoms. These symptoms must be present for at least three days per week, with onset at least six months before diagnosis and persistence over the past three months [3]. The Rome IV criteria revised and simplified the definition, describing FD as the presence of postprandial fullness, early satiation, epigastric pain, or burning, in the absence of any structural disease that can explain the symptoms, with symptom onset at least six months prior and active symptoms during the last three months [4]. Due to the absence of an identifiable organic cause, the variability of symptoms, and significant individual differences, FD presents a clinical challenge in both diagnosis and treatment [5,6].

Palpation is the primary method used in AEs, widely practiced in TEAM and Kampo medicine in Japan. TEAM doctors diagnose abdominal signs such as stiffness, coldness, and fullness with discomfort in the chest and hypochondrium (FDCH), which are key indicators of pathological changes in the body [1,2,7,8,9,10,11]. AEs broadly evaluate abdominal shape, color, and sounds [7]. Among these, FDCH is a commonly observed symptom in FD in TEAM clinics. Han et al. [12] identified liver depression with qi stagnation and spleen deficiency as the most frequent pattern in FD, typically accompanied by FDCH [13,14]. This diagnostic approach reflects the diverse educational backgrounds of TEAM practitioners: in Japan, TEAM is primarily practiced by general physicians trained in Western medicine who incorporate Kampo into their clinical practice; whereas in Korea and China, TEAM is delivered by licensed practitioners who complete over six years of specialized education in traditional medicine colleges, typically including internships and clinical residencies.

FDCH-related disease codes are recognized in the ICD-10 and ICD-11, officially adopted at the World Health Assembly [15,16]. These codes include unspecified chest pain, other digestive system symptoms, and FD as shown in Table 1. To diagnose FDCH, TEAM doctors assess abdominal shape, stiffness, and tenderness [17,18], though these methods remain subjective and qualitative in nature.

In this prospective observational feasibility study, we aimed to explore potential metrics for quantifying FDCH and assess the consistency between FDCH diagnoses made by TEAM doctors and objectively measurable abdominal signs. To achieve this, we conducted clinical studies using specialized diagnostic devices designed for precise abdominal measurements. We hypothesized that significant differences exist in abdominal features between individuals with and without FDCH. Our objective was to identify physical metrics that clearly distinguish individuals with FDCH from those without.

## 2. Methods

### 2.1. Candidate Physical Properties

The TEAM doctor was positioned on the right side of the patient. The patient lay supine on the bed with both legs extended and was given at least 5 min of rest before examination while the whole body was relaxed. In FDCH, fullness and discomfort in the abdomen are related to shape and pain. Therefore, the TEAM doctors measured abdominal shape, stiffness, and tenderness. These procedures constitute the AE.

In this study, we only used devices for measuring geometry, indented depth, and pressure to extract quantitative indices. Therefore, we reviewed a few previous studies as follows. External beam irradiation for precise positioning of the target in the treatment planning coordinate system was used to create a 3D model of the patient’s surface [19]. Spiral-CT data and opto-electronic plethysmography were used to obtain sternal angle and sternal and sternocostal kinematics in supine position and thoraco-abdominal surface shape during breathing, respectively [20,21]. The following studies investigated the depth and pressure at which pain was felt [22,23] at specific acupuncture points [18,24] for stiffness and tenderness.

**Figure 1:**
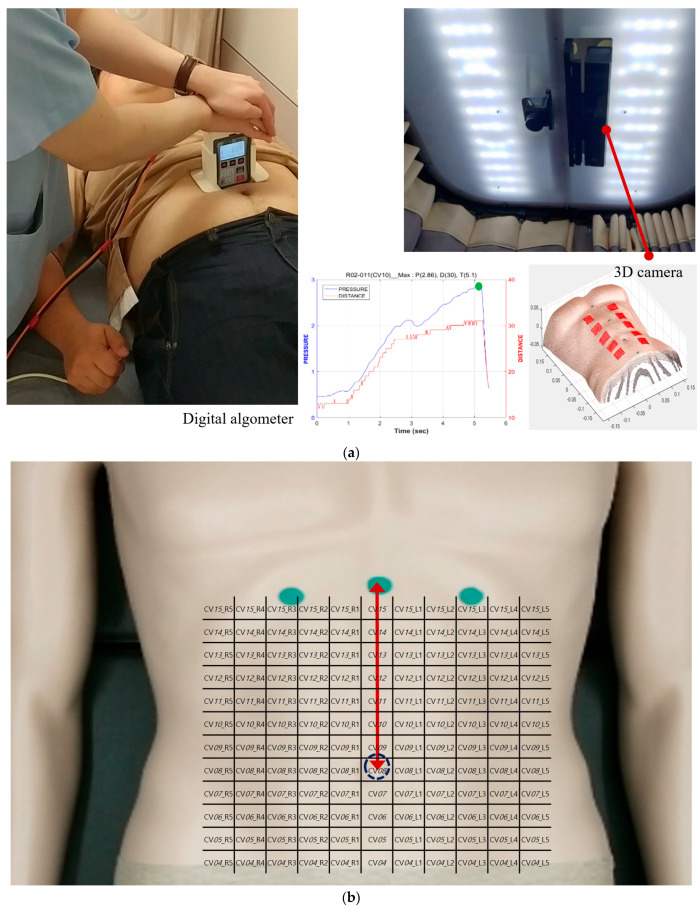
Measurement on the lattices with the devices: (**a**) two devices, and (**b**) measurable lattices with three marked points and a navel on the abdomen.

Since FDCH requires a comprehensive diagnosis that combines three diseases, such as ICD-11, unlike previous studies, the geometry of the abdomen was assessed using a 3D camera (Microsoft Kinect v2, Microsoft Inc., Redmond, Washington, USA), and the painful area and resistance of the abdomen were measured with a digital algometer combining a digital force gage (FPIX, Wagner instruments, Greenwich, CT, USA) and a laser distance sensor (OWRB4040 AAS1, Welotec, Laer, Germany) in an external housing as shown in Figure 1a. The repeatability of the indicators with each device was confirmed [25]. The depth sensor in Kinect v2 adopts a method called Time of Flight (TOF), which obtains depth information from the time when the emitted infrared rays are reflected and return. This results in a ground sample distance (GSD) between 1.4 mm at 0.5 m range and 12 mm at 4.5 m range [26], which is depth resolution. Color spatial resolution is 1920 × 1080 pixels and acquisition rate is 30 Hz [27]. The patient-report pressure algometer was improved as a digital algometer [28]. The abdominal contact form was made of aluminum with a rounded corner, 40 mm wide and 10 mm long, and the part that contacts the skin was made of 2 mm thick rubber material, which can be pressed like a human finger. A laser sensor was connected to the external housing to measure the pressure depth on the abdomen. It determined that FD and abdominal stiffness had significant association at 2 acupoints, whereas FD and abdominal tenderness had significant association at all 12 acupoints [28].

The examiner touched the subject’s upper abdomen with their index finger and gradually moved it up to the upper apex along the median line. The finger was first felt at the point where the sternum and both ribs meet, which is known as the xiphisternum or Jungjeong (CV*16*), and then the examiner attached green marker 1 there as shown in Figure 1b. A red arrow line is indicated between the xiphisternum and the navel, showing the region that was divided into 8 equal parts to define 1 chon. The navel’s boundary was depicted as a dotted curve. The abdomen was then partitioned at intervals of 1 chon, as depicted in Figure 1b, and the navel was denoted by a dotted circle. Hence, the measurements were taken at the segmented points on the abdomen, as demonstrated in Figure 1b. Because chon is a measure proportional to body size, it cannot be expressed in SI units. Green markers 2 and 3 were located at two points where the ribs meet at a distance of two chons to the left and right of the central vertical axis with the xiphisternum. Together, the markers formed a sterno-costal angle.

The points that were partitioned are as follows: the center points were the acupuncture points from CV*15* to CV*4*, and the points CV*N*_L1, CV*N*_L2, CV*N*_L3, CV*N*_L4, and CV*N*_L5 points were located 1, 2, 3, 4, and 5 chons to the left of the CV*N*, respectively (where *N* = 15 to 4). Similarly, the points CV*N*_R1, CV*N*_R2, CV*N*_R3, CV*N*_R4, and CV*N*_R5 points were located to the right of the CV*N*.

A 3D camera was used to capture an image with depth information and to divide the image into a lattice. The indented depths and pressures at the same sites pressed in the AE were evaluated by proficient TEAM doctors using the digital algometer. Both the subject and the examiner maintained the same AE posture as examining the existence of abdominal stiffness and tenderness. The examiner held the device vertically against the subject’s abdomen, and gradually applied pressure corresponding to the graph displayed on the monitor at a rate of 1 kg/cm^2^/s. The subject was instructed to immediately press the buzzer when they started to feel pain. When the buzzer was pressed, the operator immediately stopped applying pressure, and the indented depth and pressure were automatically recorded on the monitor. These procedures were repeated at thirteen points: CV*14* (CV14), CV*13*_L2 (left ST20), CV*13*_R2 (right ST20), CV*12* (CV12), CV*12*_L2 (left ST21), CV*12*_R2 (right ST21), CV*10* (CV10), CV*08*_R2 (right ST25), CV*08*_L2 (left ST25), CV*06* (CV6), CV*04* (CV4), CV*04*_L2 (left ST28), and CV*04*_R2 (right ST28) [24]. These acupuncture points were selected based on a previous review paper on AEs [18].

### 2.2. Participants

We performed a Power analysis to determine the sample size for our FD patient group and control group. This analysis was conducted using an α error probability of 0.05, a Power (1-β error probability) of 0.8, an allocation ratio of 1, and an independent samples *t*-test. By comparing the required sample sizes for Cohen’s d effect sizes between 0.5 and 0.6, we decided on a sample size of 60 participants for both the test and control groups.

In total, 63 participants from the age of 20 to 65 years with FD and complaints of epigastric discomfort or pain and 60 healthy participants without any digestive problems were screened at Kyung Hee University Hospital in Gangdong from April 2018 to July 2020. The participants with FD fulfilled the Rome III diagnostic criteria for FD [29]. Patients diagnosed with organic diseases such as erosive esophagitis, peptic ulcer, dysplasia, lymphoma, esophageal cancer, and gastric cancer through esophagogastroduodenoscopy were not included in the study. Also, patients who exhibited specific clinical symptoms of irritable bowel syndrome or alarm symptoms like weight loss, black stool, and dysphagia were excluded. Since FD symptoms originate in the upper gastrointestinal tract and are not related to structural issues in the colon, colonoscopy, which is a procedure specifically designed to examine the large intestine, does not provide direct diagnostic information for FD. Therefore, we did not confirm its performance [4,29]. In addition, participants who had a history of mental illness or digestive surgery, or were pregnant or breastfeeding, were excluded from the study. Those who had taken part in another clinical study within the previous month, had HIV, or faced challenges in participating in the study (such as paralysis, severe drug addiction, scheduling conflicts, severe vision or hearing impairments, and illiteracy) were also excluded. Participants who were considered healthy and met the exclusion criteria for the FD group, and who had an overall score of less than 20 on the visual analog scale (VAS, 0–100), were included in the study. The Institutional Review Boards of Kyung Hee University Hospital of Gangdong approved the study and provided ethical approval (approval ID: KHNMCOH 2018-03-002-001) on 11 April 2018, and it was conducted according to the guidelines for good clinical practice established by the International Conference on Harmonization. Informed consent was obtained from all participants following a full explanation of the purpose of the study. Decisional capacity was required for informed consent, and participation was voluntary after the details of the study were provided. Adverse events were recorded in detail in the case report form. No dropouts were recorded during the study. The registration number of the clinical trial was KCT0003369 and it was registered on 23 November 2018.

In this clinical study, abdominal examination (diagnosis) by TEAM doctors was performed to diagnose abdominal characteristics. A questionnaire about pattern identification for FD [30] and the Nepean dyspepsia index—Korean version [31] written for Korean medicine doctors were used to identify FD and its patterns. The questionnaires to check the patient’s symptoms included a Phlegm Pattern (Damum) questionnaire [32], food retention questionnaire [33], spleen qi deficiency questionnaire [34], scale for stomach qi deficiency pattern [35], cold-heat questionnaire [36], and deficiency-excess questionnaire [37]. A TEAM doctor diagnosed FDCH strictly by combining the above characteristics. A gastric emptying test using ultrasonography, abdominal sound evaluated by stethoscope, and abdominal wall thickness evaluated by sonography were not analyzed due to insufficient data.

The TEAM doctors evaluated the subjects’ abdominal stiffness and tenderness by examining their abdomen with their right index, middle, and ring fingers, using the diagnostic methods described in previous studies [22,23], which constitute abdominal examination. They applied slow pressure to the patient’s abdominal acupoints to determine the presence of abdominal stiffness and tenderness.

Three TEAM doctors with more than 2 years of clinical experience independently examined the participants in separate offices with a temperature of 18 °C and humidity of 40~50%. The standard AE process was followed, and the TEAM doctors were well trained before the initiation of the study. The final diagnosis of abdominal signs was determined by the consensus of 2 out of 3 TEAM doctors, which is considered the gold standard.

### 2.3. Calculation of Quantitative Physical Factors

Quantitative physical factors were calculated using the data recorded by the measurement devices. The values acquired from the digital algometer were the pressure (Pr(pos(x,y))), the indented depth ($d_{i}(pos(x,y))$), and the pressure per unit depth (stiffness: stiff(pos(x,y))) at the moment when the patient felt pain at the acupuncture point. The stiffness equation is as follows:

(1) $$stiff\left(pos(x,y)\right)=\frac{Pr(pos(x,y))}{d_{i}(pos(x,y))}$$

Several geometric features, such as the distance between two specified acupuncture points of CV*N*_L2 and CV*N*_R2 ($dis\left(y\right)$), sterno-costal angle, depth difference across the rib region at markers 2 and 3, depth value (dep(pos(x,y))), depth difference between two acupuncture points for checking horizontal symmetry (${∆}_{dep}\left(x,y\right))$, and angle difference between the surface normal vectors at two acupuncture points (${∆}_{angle}\left(pos\left(x,y\right),pos(x,y+1)\right)$) were assessed as shown in Equations (2)–(5). The measured points used for acquiring the geometric information belonged to the points in Figure 1b. The $pos\left(x,y\right)$ is the location to get a feature.

(2) $$dis\left(y\right)=\left|CVy\_R2-CVy\_L2\right|$$

(3) $$pos\left(x,y\right)=\left\{\begin{array}{c} CVy\_Rx,\begin{array}{cc} \; & right\;position \end{array}\\ CVy,\;\;\;\;x=0\;\;\;\;\\ CVy\_Lx,\begin{array}{cc} \; & \;left\;position \end{array} \end{array}\right.$$

(4) $${∆}_{dep}\left(x,y\right)=\left|dep\left(CVy\_Rx\right)-dep\left(CVy\_Lx\right)\right|$$

(5) $${∆}_{angle}\left(pos\left(x,y\right),pos(x,y+1)\right)=\cos^{-1}\frac{\vec{N}\left(pos\left(x,y\right)\right)\cdot \vec{N}\left(pos\left(x,y+1\right)\right)}{\left|\vec{N}\left(pos\left(x,y\right)\right)\right|\left|\vec{N}\left(pos\left(x,y+1\right)\right)\right|}$$

where $\vec{N}\left(pos\left(x,y\right)\right)$ is the surface normal vector at $pos\left(x,y\right)$.

### 2.4. Statistical Analysis

All statistical analyses were performed using R version 4.0.0 for Windows, and a significance level of α = 0.05 was used. The differences between groups according to AEs performed by TEAM doctors in the general characteristics were assessed using independent two-sample *t*-tests for the continuous variables and chi-squared tests for the categorical variables.

General linear models were used to compare the differences in variables from the devices between groups, adjusted for several covariates, such as sex, age, BMI, alcohol, and caffeine consumption. The marginal means of each group were estimated by fitting general linear models adjusted for covariates. For variables with missing value, complete case analyses were conducted.

## 3. Results

### 3.1. Comparison of General Characteristics

A total of 123 participants were screened for this study, with 2 failing to meet the inclusion criteria and 1 participant withdrawing consent. Critically, there were no dropouts during the study, leading to 120 participants completing the study. While this sample size, unevenly classified into FDCH (20 participants) and non-FDCH (100 participants) groups, would be insufficient for robust statistical significance testing in a main study, it adequately serves as a feasibility study. Table 2 presents the differences between the two groups, classified by abdominal examinations (AEs) performed by TEAM doctors. We found no significant differences in demographic characteristics (age, body temperature, sex, alcohol intake, or caffeine intake) between the FDCH and non-FDCH groups. Importantly, Table 3 demonstrates a strong association between FD and FDCH patients, indicating that most FDCH patients belonged to the FD group, with this association being statistically significant.

Our study participants included individuals who smoked either conventional cigarettes or electronic cigarettes with nicotine, who were all counted as smokers. Of the total, 12 participants were smokers and 108 were non-smokers. The correlation between smoking status and FDCH was found to be 0.01436.

### 3.2. Comparison Between Groups According to the Abdomen Examinations Performed by TEAM Doctors

Among the physical factors obtained from two devices, the physical factors significantly associated with FDCH were the algometric and geometric factors. Table 4 shows statistically significant differences in pressure, depth, and the pressure-to-depth ratio at which pain was felt between the groups with and without FDCH, which were algometric features. The units of pressure and depth were kgf/cm^2^ and mm, respectively. Since the measured time can depend on how the assessor presses the abdomen, the time is not of great significance. Table 4 shows the average pressure, depth, and stiffness values of FDCH and non-FDCH, the range of depth values in parentheses, and the average value, range of difference values, and *p* value for the difference between the two groups. Across the locations, the point estimates for the mean differences between FDCH and non-FDCH in the algometric factors of pressure, depth, and stiffness range from −1.47 to −0.86, −8.75 to −4.46, and −0.31 to −0.12, respectively.

Regarding the geometric information, the following measurements were found to be larger in the presence of the FDCH: the distance between CV*08*_R2 and CV*08*_L2 (FDCH: 77.3 mm [70.64, 83.97], non-FDCH: 68.28 mm [63.98, 72.58]), the sterno-costal angle (FDCH: 113.7 degrees [104.63, 122.76], non-FDCH: 98.06 degrees [92.21, 103.91]), and the depth differences across the rib region at markers 2 and 3 (FDCH: 4.69 mm [3.75, 5.63], non-FDCH: 3.69 mm [3.08, 4.29]).

The depth differences between around CV*11*_R2 and around CV*11*_L2 were smaller in the presence of FDCH than in its absence (FDCH: 1.07 mm [0.3, 1.84], non-FDCH: 2.16 mm [1.67, 2.66]). The angle differences between the surface normal vectors around CV*14* and CV*15* (FDCH: 5.85 degrees [3.3, 8.41], non-FDCH: 8.63 degrees [6.98, 10.28]) and those of around CV*14*_L1 and CV*15*_L1 (FDCH: 13.67 degrees [−1.03, 28.36], non-FDCH: 30.75 degrees [21.27, 40.24]) were smaller in the presence of FDCH. The angle differences between the surface normal vectors around CV*09*_L2 and CV*10*_L2 were larger in the presence of FDCH (FDCH: 9.23 degrees [6.69, 11.77], non-FDCH: 6.24 degrees [4.6, 7.88]). The depth values at CV*09*_L1, CV*10*_L2, CV*11*_L1, CV*11*_L2, CV*12*_L1, CV*13*, CV*13*_L1, CV*13*_L2, and CV*14*_L1 were smaller in the presence of FDCH, as shown in Table 5. The standard depth value was 0, and a negative depth value indicated a position lower than 0. The lower the position was, the smaller the depth value. Table 5 shows the average depth values of FDCH and non-FDCH, the range of depth values in parentheses, the average value and range of difference values, and the *p* value for the difference between the two groups. The point estimates for the mean differences in the geometric factor of depth between FDCH and non-FDCH across the locations range from −2.09 to −1.66.

## 4. Discussion

A previous study [22] found that the algometer had high sensitivity and specificity in producing pressure depth and pressure pain threshold (PPT) for diagnosing abdominal stiffness and tenderness. The PPT validity at CV*14* was found to have a sensitivity of 73.1%, a specificity of 77.8%, and an AUC of 0.807 with a *p*-value of <0.001. Additionally, the standard deviations of repeated measurements of geometric information from 10 regions using a 3D camera were between 0.31 and 0.81 mm, indicating high repeatability [25]. As the reliability of the devices was confirmed, the measurement indices were considered reliable.

**Figure 2:**
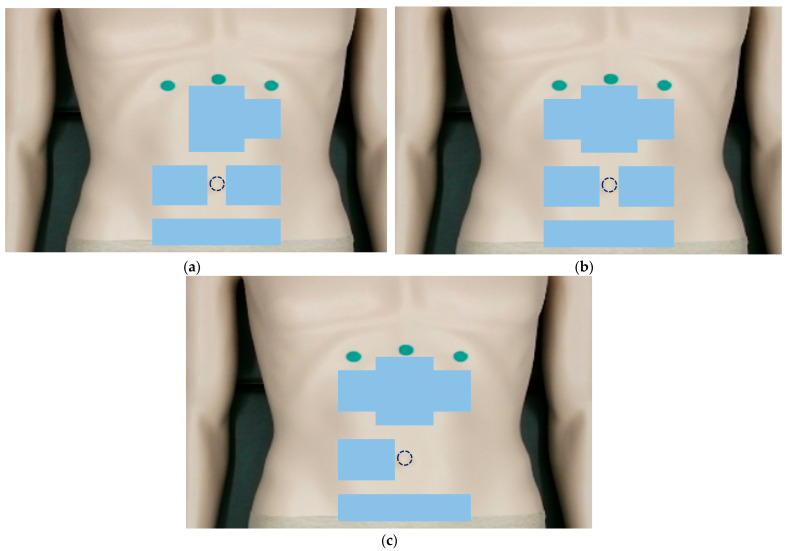
Acupuncture points with significant differences between the people with and without fullness and discomfort in the chest and hypochondrium (FDCH): (**a**) the ratio of pressure to depth (stiffness), (**b**) pressure, and (**c**) depth.

In clinical practice, patients with FDCH have a fuller upper abdomen and flank compared with those who have no symptoms. Therefore, when the TEAM doctors used the index, middle, and ring fingers to push upward from the lower edges of the costal arches on both sides, they felt resistance in the fingers, and the patient expressed pain. Figure 2 shows the mapping of a 3 × 3 window over a stochastically significant acupoint (*p* < 0.05) for (a) the pressure-to-depth ratio, (b) pressure, and (c) depth, which are algometric features related to FDCH as described in Table 4. The blue color indicates that values in individuals with FDCH are lower than those in the control group, whereas the orange color indicates the opposite. When measuring abdominal pain and resistance using a digital algometer, overall, physical algometric values decreased in the presence of FDCH, indicating decreased abdominal elasticity and stiffness, and increased sensitivity to external pressure.

**Figure 3:**
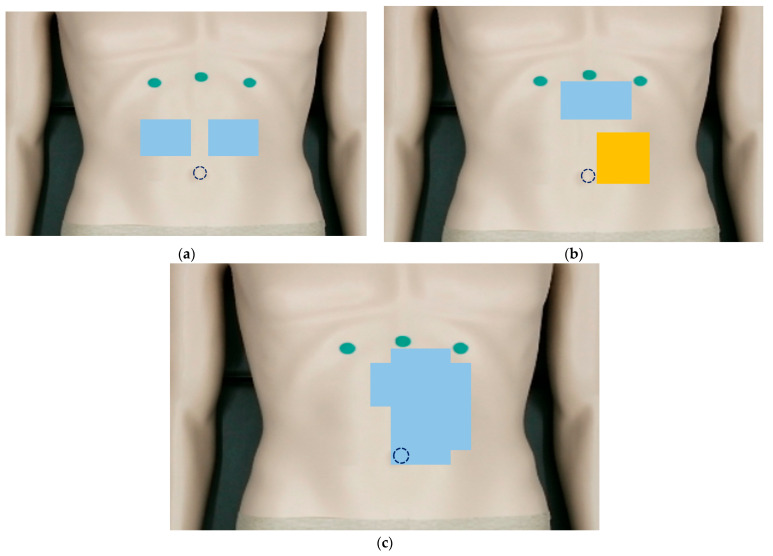
Colored points with significant differences between the groups with and without fullness and discomfort in the chest and hypochondrium (FDCH): (**a**) depth difference, (**b**) angle differences of the surface normal vectors, and (**c**) depth.

The FDCH patients had a longer distance between CV*08*_R2 and CV*08*_L2, a larger sterno-costal angle, and a fuller costal arch in the right rib region. Figure 3 depicts the mapping of a 3 × 3 window over a significant acupoint (*p* < 0.05), regarding the abdominal geometric information assessed using the 3D camera. The blue color indicates that values in individuals with FDCH are smaller than those in the control group, while the orange color indicates the opposite. When a significant difference was observed (*p* < 0.05), Figure 3a the depth differences around CV*11*_R2 and CV*11*_L2 were smaller, indicating that the center region had a flatter shape. Figure 3b shows the angle differences between the surface normal vectors around CV*14* and around CV*15* and those around CV*14*_L1 and around CV*15*_L1 were smaller, indicating that the upper region was even. However, the left central region was bumpy around CV*10*_L2 and CV*9*_L2. In Figure 3c, the depth values at CV*09*_L1, CV*10*_L2, CV*11*_L1, CV*11*_L2, CV*12*_L1, CV*13*, CV*13*_L1, CV*13*_L2, and CV*14*_L1 were lower as described in Table 5. Therefore, the abdominal shape tends to be flat or droop rather than appear convex on the upper surface. Since an impaired antro-fundic reflex in patients with FD can provoke defective gastric accommodation, leading to an altered distribution of gastric contents and antral overload [38], it is thought that the altered distribution can cause the ruggedness around CV*10*_L2 and CV*9*_L2 where the gastrointestinal tract is located, which requires more research. These evaluations with quantitative AE methods revealed signs of FDCH, as diagnosed by the TEAM doctors. Therefore, the algometric and geometric metrics can be applied to diagnosing FDCH. Specifically, dividing the abdomen area into a lattice is an essential factor for objective measurement.

FDCH, which occurs in the upper abdomen beneath the chest, is a symptom associated with various diseases. Studies have reported that it is commonly observed in patients with inflammatory conditions such as acute acalculous cholecystitis [39], hepatitis B cirrhosis [40], and inflammatory bowel disease, particularly in the respiratory and upper gastrointestinal tract types [41]. Other conditions linked to FDCH include cholelithiasis [42], depressive neurosis (especially in cases of liver qi stagnation) [43], globus hystericus [44], and acute pancreatitis, particularly in chest binding and interior excess syndrome [45]. In TEAM syndrome differentiations, liver qi stagnation manifests as pain and distension in the hypochondrium, irritability, depression, breast distension and pain, dysmenorrhea, menstrual disorders, and lower abdominal pain [46]. Chest binding and interior excess syndrome is characterized by hardness, fullness, and pain in the chest, hypochondrium, and upper abdomen, often accompanied by tenderness and discomfort.

Given its association with various diseases and its clinical importance in both Western and traditional medicine, establishing objective diagnostic tools for FDCH is essential. Our measurement method offers significant advantages, being non-invasive, radiation-free, and cost-effective, which simplifies its implementation in medical settings. It allows for a comprehensive assessment of the entire abdomen’s geometric and stiffness properties to classify FDCH, holding significant potential for providing objective and quantifiable diagnostic indicators. In this study, we utilized metrics from two diagnostic devices to objectively identify significant variables that differentiate patients with FDCH from healthy controls. Moving forward, we aim to analyze abdominal symptoms in other functional and neurotic diseases to establish their respective metrics. These devices can assist in diagnosing diseases based on abdominal signs and enable pattern identification (PI) by integrating clinical data, allowing for the classification of patients into distinct groups. Through further miniaturization and lightweight design, these devices will be developed into wearable tools for remote, non-contact diagnosis.

To support this clinical implementation, the abdominal examination devices (AEDs) used in this study were developed to facilitate clinical research through the acquisition and analysis of biosignals. Accordingly, the present study holds significant clinical relevance, as it contributes to enhancing diagnostic accuracy, enabling precise monitoring of physiological responses, and informing evidence-based treatment decisions. Excluding the computer, the Microsoft Kinect v2 was originally priced at approximately $199 USD, but it has since been discontinued. As a replacement, the Intel RealSense depth camera is now commonly used, with a price of around $320 USD. The digital algometer used in this study costs approximately $1500 USD. The signal processing and analysis software was custom-developed by the research team. Once commercialized, the overall cost is expected to decrease, thereby improving the availability and accessibility of these methods in clinical settings.

While Western medicine classifies conditions using ICD-10 and ICD-11, the lack of anatomically apparent symptoms in FD often leads to pharmaceutical treatments based on trial and error. This means medication is administered to gauge its effectiveness on a patient’s symptoms before deciding on continued use or alternative options. In contrast, TEAM’s definition of FDCH allows for a more structured and predefined treatment pathway as shown in Table 6 [47,48,49,50].

## 5. Limitations

There are several limitations to consider when interpreting these results. First, while the dataset was sufficient to distinguish between FDCH and non-FDCH groups, it was not large enough to analyze detailed differences based on gender and age. Future studies should aim to collect a larger dataset covering a broader range of demographic characteristics, which could also be utilized for implementing deep learning algorithms. Second, since our analysis was conducted on subjects without organic abdominal diseases, the findings may not be fully generalizable to patients with such conditions. Third, the applicability of our results to different ethnic groups is uncertain, as variations in skin and skeletal characteristics may influence the findings. To address this, a larger and more diverse dataset should be gathered to better represent different ethnicities. Despite these limitations, obtaining measurable properties of functional diseases is a significant step toward quantifying abdominal examinations (AEs). This research paves the way for more precise treatment approaches, such as personalized herbal medicine and acupuncture, based on quantified symptoms of FD.

## 6. Conclusions

To date, TEAM doctors have subjectively diagnosed abdominal signs. In this article, to objectively diagnose FDCH in the abdomen, metrics obtained from two devices were used to explore the possibility of identifying significant variables that can differentiate between patients with FDCH and controls. Through the physical characteristics of each location of the abdomen, it is thought that not only abdominal symptoms but also various abdominal visceral diseases can be patterned. Therefore, recording a significant amount of clinical data is crucial. In the future, accumulating more clinical data will lead to more precise and quantitative findings related to abdominal metrics.

## Figures and Tables

**Table 1:** Disease codes associated with FDCH.

Diseases	ICD-10 Version: 2019	ICD-11
Chest pain, unspecified	R07.4	MD30.Z
Other specified symptoms or signs involving the digestive system or abdomen	R19.8	ME1Y
Functional dyspepsia	K30	DD90.3

**Table 2:** Demographic information between groups with and without fullness and discomfort in the chest and hypochondrium.

	Fullness and Discomfort in the Chest and Hypochondrium		
	Existence	Nonexistence	*p*-Value
Number of subjects	20	100	
Age (years)	36.35 ± 11.77	40.32 ± 10.97	0.176
Body temperature (°C)	36.24 ± 0.35	36.34 ± 0.36	0.256
Sex			0.443
Men	5 (25%)	15 (15%)	
Women	15 (75%)	85 (85%)	
Alcohol consumption			0.102
No	14 (70%)	47 (47%)	
Yes	6 (30%)	53 (53%)	
Caffeine consumption			0.241
No	7 (35%)	20 (20%)	
Yes	13 (65%)	80 (80%)	

All variables are summarized as the mean ± standard deviation for the continuous variables and as the frequency (percentage) for the categorical variables. The *p*-values for differences between two groups were obtained from independent two-sample *t*-tests for the continuous variables and from Fisher’s exact tests for the categorical variables.

**Table 3:** Association between FD and FDCH patients.

	FDCH	Non-FDCH	Fisher’s Exact Test *p*-Value	Total
FD group	16	44	0.006	60
non-FD group	4	56		60
Total	20	100		

FD: functional dyspepsia; FDCH: fullness and discomfort in the chest and hypochondrium.

**Table 4:** Pressure, depth, and the ratio of pressure to depth (stiffness) when the pain started.

Variables	Location	FDCH	Non-FDCH	Mean Difference (95% CI)	*p*-Value
Pressure	CV*14*	2.63 (2.1, 3.16)	3.93 (3.59, 4.28)	−1.31 (−1.87, −0.75)	<0.001
	CV*13*_R2	2.53 (2.01, 3.05)	3.95 (3.61, 4.28)	−1.41 (−1.96, −0.87)	<0.001
	CV*13*_L2	2.54 (2.01, 3.06)	3.86 (3.52, 4.2)	−1.32 (−1.87, −0.77)	<0.001
	CV*12*	2.63 (2.01, 3.24)	4.1 (3.7, 4.5)	−1.47 (−2.12, −0.82)	<0.001
	CV*08*_R2	2.34 (1.81, 2.87)	3.46 (3.12, 3.8)	−1.12 (−1.68, −0.56)	<0.001
	CV*08*_L2	2.51 (1.95, 3.07)	3.69 (3.33, 4.06)	−1.18 (−1.78, −0.59)	<0.001
	CV*04*_R2	2.24 (1.71, 2.78)	3.38 (3.03, 3.72)	−1.14 (−1.7, −0.57)	<0.001
	CV*04*_L2	2.13 (1.53, 2.73)	3.51 (3.13, 3.9)	−1.38 (−2.01, −0.75)	<0.001
	CV*04*	2.35 (1.86, 2.83)	3.2 (2.89, 3.52)	−0.86 (−1.37, −0.34)	0.001
Depth	CV*14*	29.54 (26.1, 32.98)	34.71 (32.49, 36.93)	−5.17 (−8.8, −1.54)	0.006
	CV*13*_R2	26.82 (23.05, 30.59)	35.58 (33.14, 38.01)	−8.75 (−12.73, −4.77)	<0.001
	CV*13*_L2	31.05 (27.84, 34.26)	36.11 (34.04, 38.18)	−5.06 (−8.44, −1.68)	0.004
	CV*12*	29 (26.09, 31.9)	34.66 (32.78, 36.53)	−5.66 (−8.72, −2.6)	<0.001
	CV*08*_R2	32.96 (29.63, 36.29)	37.42 (35.27, 39.57)	−4.46 (−7.98, −0.95)	0.013
	CV*04*_R2	34.73 (31.55, 37.9)	41.5 (39.45, 43.55)	−6.77 (−10.12, −3.42)	<0.001
	CV*04*_L2	34.8 (31.71, 37.89)	40.99 (38.99, 42.99)	−6.19 (−9.46, −2.93)	<0.001
	CV*04*	33.3 (29.95, 36.65)	38.84 (36.68, 41.01)	−5.55 (−9.08, −2.01)	0.002
Stiffness	CV*14*	0.88 (0.73, 1.04)	1.14 (1.04, 1.24)	−0.25 (−0.41, −0.09)	0.002
	CV*13*_L2	0.79 (0.67, 0.91)	1.07 (0.99, 1.15)	−0.28 (−0.41, −0.15)	<0.001
	CV*12*	0.88 (0.71, 1.04)	1.19 (1.08, 1.29)	−0.31 (−0.48, −0.13)	0.001
	CV*08*_R2	0.71 (0.58, 0.84)	0.94 (0.85, 1.02)	−0.22 (−0.36, −0.09)	0.001
	CV*08*_L2	0.69 (0.56, 0.81)	0.94 (0.86, 1.02)	−0.25 (−0.39, −0.12)	<0.001
	CV*04*_R2	0.64 (0.52, 0.76)	0.81 (0.73, 0.89)	−0.17 (−0.3, −0.04)	0.013
	CV*04*_L2	0.62 (0.48, 0.75)	0.86 (0.77, 0.95)	−0.24 (−0.39, −0.1)	0.001
	CV*04*	0.69 (0.59, 0.8)	0.82 (0.75, 0.88)	−0.12 (−0.23, −0.01)	0.03

**Table 5:** Depth values around locations for geometry information.

Location	FDCH	Non-FDCH	Mean Difference (95% CI)	*p*-Value
CV*09*_L1	−11.22 (−12.63, −9.8)	−9.56 (−10.47, −8.64)	−1.66 (−3.16, −0.16)	0.03
CV*10*_L2	−13.95 (−15.49, −12.41)	−12.15 (−13.14, −11.15)	−1.8 (−3.43, −0.18)	0.03
CV*11*_L1	−12.83 (−14.58, −11.08)	−10.96 (−12.09, −9.83)	−1.88 (−3.72, −0.03)	0.046
CV*11*_L2	−10.2 (−11.99, −8.4)	−8.21 (−9.37, −7.05)	−1.99 (−3.88, −0.09)	0.04
CV*12*_L1	−9.49 (−11.35, −7.62)	−7.39 (−8.6, −6.19)	−2.09 (−4.06, −0.12)	0.037
CV*13*	−9.14 (−10.73, −7.54)	−7.29 (−8.32, −6.26)	−1.85 (−3.53, −0.17)	0.031
CV*13*_L1	−9.22 (−10.92, −7.51)	−7.33 (−8.43, −6.23)	−1.89 (−3.69, −0.09)	0.04
CV*13*_L2	−9.08 (−10.89, −7.26)	−6.99 (−8.17, −5.82)	−2.08 (−4, −0.16)	0.034
CV*14*_L1	−9.64 (−11.14, −8.14)	−7.62 (−8.59, −6.66)	−2.01 (−3.6, −0.43)	0.013

**Table 6:** Etiological Types and Representative Herbal Prescriptions for FDCH.

Etiological Type	Representative Herbal Prescription	Main Effects and Indications
Liver Qi Stagnation Type	Chaihu Shugan San	Chest and flank fullness due to Liver Qi stagnation, side pain, indigestion
	Jiawei Xiaoyao San	Liver Qi stagnation with heat symptoms, irregular menstruation in women
Phlegm Retention Type	Erchen Tang (modified)	Chest and flank fullness due to phlegm, vomiting, sputum, etc.
	Xiaobanxia Jia Fuling Tang	Phlegm retention with severe nausea
Blood Stasis Type	Guizhi Fuling Wan	Chest and flank pain due to blood stasis, abdominal mass, dysmenorrhea
	Dahuang Mudanpi Tang	Severe pain with blood stasis and accompanying constipation

## Data Availability

The data that support the findings of this study are available upon reasonable request from the corresponding author.
